# The mechanism of Girdin in degenerative brain disease caused by high glucose stimulation

**DOI:** 10.3389/fendo.2022.892897

**Published:** 2022-10-03

**Authors:** Longteng Liu, Jinsong Zhang, Yanxi Han, Dongge Liu

**Affiliations:** ^1^ Department of Pathology, Beijing Hospital; National Center of Gerontology; Institute of Geriatric Medicine; Chinese Academy of Medical Sciences, Beijing, China; ^2^ National Center for Clinical Laboratories, Beijing Hospital, National Center of Gerontology; Institute of Geriatric Medicine, Chinese Academy of Medical Sciences, P.R. China; Graduate School of Peking Union Medical College, Chinese Academy of Medical Sciences, Beijing, China

**Keywords:** Girdin, menstrual degenerative disease, diabetic encephalopathy, Akt, STAT3

## Abstract

Girdin, as an actin-binding protein, plays a major role in maintaining the stability of the actin skeleton structure and affects the growth, development, and migration of neurons. This study discusses the mechanism of Girdin in brain degeneration caused by high glucose stimulation. We examined the expression of Girdin in diabetic patients. The positive expression rate of Girdin in the diabetic group was 17.2% (5/29), which was obviously lower than the positive expression rate of 83.3% (20/24) in the non-diabetic group. We examined the expression of Girdin and its signaling pathway-related proteins Akt and STAT3 in hippocampal neurons induced by high glucose. The results showed that, in contrast to the control group (glucose concentration = 25 mmol/L), the expression of Girdin in the high-glucose group (glucose concentration = 225 mmol/L) was reduced (*P* < 0.05); the phosphorylation levels of Akt and STAT3 related to Girdin signaling pathway were also reduced (*P* < 0.05). Under high-glucose stimulation, the structure of neurons is abnormal, such as the reduction or disappearance of dendritic spines, and the number of neurons is reduced. In addition, Girdin and Akt were less expressed in neurons and synapses, especially the most obvious reduction in synaptic terminals. The activity of Girdin and its signaling pathway-related proteins Akt and STAT3 decreased in neurons under high glucose stimulation, indicating that the mechanism of Girdin in brain degeneration caused by high glucose stimulation was closely related to the Akt and STAT3 pathways.

**Graphic Abstract:**

The mechanism of Girdin in degenerative brain disease caused by high glucose stimulation. This article discusses the mechanism of Girdin in brain degeneration induced by high glucose stimulation. The expression of Girdin in the diabetic group was significantly lower than that in the non-diabetic group. The expression of Girdin and its signaling pathway-related proteins Akt and STAT3 in hippocampal neurons was significantly reduced under high glucose stimulation. Under high glucose stimulation, the structure of neurons is abnormal and the number decreases; synapses become shorter. It indicates that the mechanism of brain degeneration caused by high glucose stimulation by Girdin is closely related to the Akt and STAT3 pathways.

## Graphical Abstract

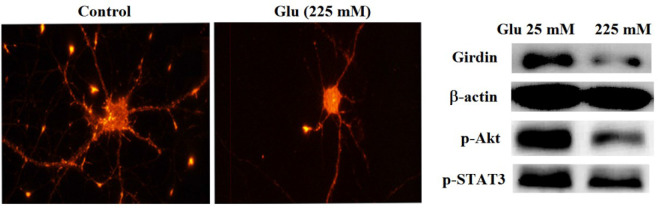

## 1 Introduction

Neurodegenerative disease (NRD) is a group of diseases caused by the chronic and progressive degeneration of nerve tissue. It is caused by degeneration, apoptosis, and loss of neurons in the brain or spinal cord (1, 2). There are two main pathological changes: one is that there is an obvious decrease in the number of neurons in the nervous system, and the other is that the structure and the function of neurons are degenerating (3–6).

In recent years, scientific interest for the degeneration of brain tissue caused by diabetes mellitus (DM) has been growing. DM is a metabolic syndrome characterized by chronic hyperglycemia, which can cause multiple organ damage and functional failure, leading to a variety of complications (7–9). Studies have shown that DM can cause changes in brain structure or neurophysiology, leading to a decline in cognitive function, which is called diabetic encephalopathy (10–12). Therefore, actively exploring the mechanism of diabetic nervous system degeneration and its prevention and treatment strategies has important medical and social significance. At present, the international community has reached a consensus on the cognitive dysfunction of the nervous system caused by diabetic encephalopathy, but its mechanism of action is still unclear. A growing body of research suggests that an important part of diabetic encephalopathy is that chronic hyperglycemia leads to hippocampal neuronal apoptosis and decreased synaptic plasticity (13–16), and neuronal synapses (branch) decrease (17, 18).

Girdin plays a major role in the nervous system. Girdin is commonly expressed in tissue cells, of which the central nervous system, kidney, and testicular tissues are the most abundant (19). Girdin interacts with the DISC1 gene and plays an important role in the growth, development, and migration of neurons. When RNA interference Girdin is missing, it will inhibit the DISCl/Girdin interaction, leading to the migration and mislocation of granular cells in the hippocampal dentate gyrus, thus causing structural damage to the hippocampal dentate gyrus neuronal cells, and axon growth defects (20). After Girdin knockout, the development of the central nervous system of mice was obviously affected. The structure of the nerve cell layer in the hippocampus was disordered, neurons lost polarity, and the formation of axons and dendrites was obviously reduced (21). It can be seen that, when Girdin is reduced or absent, it will affect the growth of neuronal cells and the development of synapses, leading to the destruction of neuronal structure and the defects of axon and dendritic growth. In summary, Girdin may be related to neuronal damage caused by degenerative diseases of the nervous system. However, so far, whether Girdin is expressed in the brain tissue of NRD-diabetic patients and whether Girdin is related to neuronal damage caused by NRD are still unclear.

In this study, we first detected the expression of Girdin in the brain tissues of diabetic patients by immunohistochemistry and analyzed the correlation between Girdin and diabetic patients; then, under the stimulation of high glucose, we established the hippocampal neuron damage model of newborn mice and observed Girdin. We further explore the relationship between Girdin and NRD-neuronal damage and its mechanism of action and provide a new idea for the pathogenesis and treatment of NRD-neuronal damage.

## 2 Materials and methods

### 2.1 Research objects

#### 2.1.1 Human autopsy specimens

The autopsy cases from the Department of Pathology of Beijing Hospital from January 1988 to December 2013 were collected. All of them were diagnosed as diabetes by the American Diabetes Association Diabetes Diagnostic Standards during their lifetime, and they were regarded as the diabetes group. There were 29 cases in the diabetes group, including 25 male and four female patients, aged between 52 and 102 years, with an average age of 84.7 ± 9.7 years. At the same time, autopsy specimens of the same age as the diabetes group were selected as the control group, and the clinical diagnosis and autopsy confirmed that they died of other diseases. There were 24 cases in the control group, including 18 male and six female patients, aged between 73 and 102 years old, with an average age of 84.0 ± 8.2 years. There was no blood relationship between the two groups of subjects, and there was no obvious difference in age (*P* > 0.05).

#### 2.1.2 Experimental animal specimens

C57 newborn mouse within 24 h of birth, both male and female, were provided by the Experimental Animal Center of Beijing Medical University.

### 2.2 Processing of autopsy specimens

The hippocampus, superior temporal lobe, amygdala, frontal lobe (anterior central gyrus), middle occipital lobe, and inferior parietal lobe were collected from the left cerebral hemisphere. The specimen was fixed with 4% formaldehyde solution, dehydrated, immersed in wax, and embedded in paraffin, and then the tissue was cut into 5-μm slices. Six sections [hippocampus, superior temporal lobe, amygdala, frontal lobe (anterior central gyrus), middle occipital lobe, and inferior parietal lobe] of each brain region were examined in each subject. The number of slices per brain region was equal in controls and DM individuals.

### 2.3 Immunohistochemical staining

The paraffin sections were deparaffinized into water. The slices were soaked in 90% formic acid for 5 min, put into the EDTA repair solution with pH = 9.0, placed in the microwave for surface antigen thermal repair for 10 min, kept there for 20 min, and taken out to cool to room temperature. The slices were added with 100 μl H_2_O_2_ for 10 min and rinsed with phosphate-buffered saline (PBS). The slices were added with 100 μl goat serum to react for 10 min. After removing the serum, the slices were added with 100 μl primary antibody, anti-Aβ (1:200, Cell Signaling, USA), and anti-Girdin (1:100, Santa Cruz, CA, USA), allowed to react for 75 min, and washed with PBS. The slices were added with 100 μl secondary antibody to react for 15 min and rinsed with PBS. The slices were added to 100 μl of freshly prepared DAB solution until the color developed. Then, these were rinsed with running water, re-stained with hematoxylin, and faded with hydrochloric acid and ethanol; 0.1% ammonia water (30 s) was used for bluing, and running water was used for rinsing. After the gradient alcohol was dehydrated, it was transparent with xylene. Finally, the piece was sealed with neutral gum. The abovementioned PBS rinses were done three times (5 min each time).

When brown–yellow particles appear in the cytoplasm and (or) nucleus, it is considered Girdin-positive, and it is judged according to the staining intensity and the percentage of stained cells, where no, light, medium, and strong staining are 0, 1, 2, and 3 points, respectively: 1—the stained cells are less than or equal to 25%, 2—the stained cells are 26 to 50%, and 3—the stained cells are greater than 50%. Thus, total score = staining intensity of each section × percentage of stained cells, where zero to one was classified as negative (–), two to three was classified as weakly positive (+), four to five was classified as moderately positive (++), and greater than or equal to 6 was classified into strong positive (+++) (21).

### 2.4 Primary culture of neurons

At 12 h before cell culture, 96-well cell culture plates (used for MTT detection), 24-well cell culture plates (used for cell fluorescence), and six-well cell culture plates (used for cell fluorescence) were coated with 0.1 mg/ml polylysine (Western blot), placed in 37°C and 5% CO_2_ incubator, and rinsed with ultrapure water before use. C57 newborn mouse within 24 h of birth were selected, and the skin was disinfected with 75% ethanol. The entire brain was taken out, placed in a Petri dish containing D-Hank’s solution, and washed three times to remove surface blood stains. The cerebral cortex was separate under the microscope, and the pia mater was removed. The hippocampus tissue was transferred into a vessel containing D-Hank’s solution. The tissue was cut into pieces and washed three times with D-Hank’s solution. Furthermore, 1 ml of 1% pancreatin and 3 ml of D-Hank’s solution were added to the shredded tissue, and this was transferred to a centrifuge tube. The centrifuge tube was digested in 37°C water bath for 15 min and shaken every 3 min. The centrifuge tube was digested with 10% serum-containing DMEM to terminate the digestion, and an appropriate amount of DNases was added to digest the DNA of the broken cells, and the tube was pipetted, in turn, until the tissue mass disappeared. The single-cell suspension was filtered with a sieve, and the cell suspension was transferred into a centrifuge tube and centrifuged at 1,000 rpm for 5 min. The supernatant was discarded, 2 ml of 10% serum DMED was added to the centrifuge tube to resuspend the cells, and the cells were inoculated in a dish (the number of cells in the 96-well plate is 2 × 10^4^ cells/ml, and the number of cells in the 24-well plate and the 6-well plate is 5 × 10^5^/ml) diluted with 10% serum in DMEM, and placed in 37°C and 5% CO_2_ incubator for culture. After 4 h, the DMEM medium was changed to Neurobasal medium to continue the culture, and then half of the medium was changed every 3 days. When the neurons were cultured to the 7th day, the Neurobasal medium with final concentrations of 25, 100, and 225 mmol/L glucose was used to change the medium, and the cell morphology and growth were observed under an inverted phase-contrast microscope. Additional tests were performed at 24, 48, and 72 h after fluid replacement.

### 2.5 Neuron experiment grouping

The cells were divided into nine groups (**Table 1**) and treated with glucose concentrations in three groups: blank control group (25 mmol/L), medium-glucose group (100 mmol/L), and high-glucose group (225 mmol/L). The treatment time for the 3 groups was 24, 48, and 72 h, respectively.

**Table 1:** Experimental grouping.

Group	1	2	3	4	5	6	7	8	9
Time (h)	24	24	24	48	48	48	72	72	72
Glucose concentration	25	100	225	25	100	225	25	100	225

### 2.6 Measurement of neuronal viability

The hippocampal neurons were inoculated into 96-well plates at 2 × 10^4^ cells/well and grouped according to different experimental treatments. Each group had four to five multiple wells. At 4 h before the termination of the culture, 20 μl of MTT solution (5 mg/ml) was added to each culture well of the cells to be tested, and the culture was continued for 4 h in an incubator at 37°C. The culture was terminated, and the medium in the culture was fully pumped out. Then, 150 μl of dimethyl sulfoxide was added to each well and shaken for 10 min to fully dissolve the crystals, and the optical density of each well was measured at a wavelength of 490 nm with a microplate reader (Bio-Rad, USA). The cell-free well was used as blank control, with zero adjusted by blank control well during color comparison.

### 2.7 Western blot detects protein expression

Neuronal cells were added to the cell lysate, and the cells were sonicated with an ultrasonic cell disruptor three times (5 s each time) and centrifuge at 13,000 rpm for 15 min at 4°C. The supernatant was collected, and 5 μl was saved to determine the protein concentration. The remaining supernatant was added to 5 × SDS-PAGE loading buffer (Sigma, USA) and heated at 100°C for 5 min to completely denaturate the protein. The concentration of the separation gel was determined according to the desired molecular weight of the target protein (Girdin protein 8% separation gel, Akt, p-Akt, STAT3, p-STAT3, and β-actin 12% separation gel). The prepared gel was installed on the electrophoresis rack. Each well was loaded with 20 μg, and protein markers (All-Chemical Gold) were added to one well. The electrophoresis device was connected, and the voltage was adjusted to 100–120 V after the sample and the concentrated gel entered the separation gel. After the electrophoresis was completed, the gel was removed and transferred to a polyvinylidene fluoride (PVDF) membrane. The PVDF membrane was soaked in 5% BSA blocking solution and gently shaken at 25°C for 1 h. Girdin (1:1,000, Santa Cruz, CA, USA), Akt (1:1,000, Cell Signaling, USA), and p-Akt (1:1,000, Cell Signaling, USA) were added with 5% skimmed milk powder blocking solution in a certain proportion. STAT3 (1:1,000, Cell Signaling, USA) and p-STAT3 (1:1,000, Cell Signaling, USA) antibodies were diluted. According to the molecular weight of the target protein, the transfer membrane was cut and placed in the primary antibody. The transfer membrane was incubated on a shaker at 4°C for 12 h and washed with 1× TBST. The second antibody was diluted with a blocking solution, and the transfer membrane was incubated with the second antibody. The membrane was placed on a shaker at 25°C for 1 h and washed with 1× TBST. Finally, chemiluminescence immunoassay was performed. Image J software was used to analyze the optical density of the band, and the ratio of the intensity of the target band to the internal reference β-actin was used as the result. All experiments were repeated three times.

### 2.8 Cellular immunofluorescence method to detect protein expression

Neuronal cells were seeded in a 24-well plate at 2 × 10^5^ per well. The cells were fixed with 4% paraformaldehyde (Sigma-Aldrich, USA) at 25°C for 20 min and washed with PBS. The cells were immersed in 0.1% TritonX-100 (Sigma-Aldrich, USA), incubated at 25°C for 10 min, and then washed with PBS. The cells were incubated with 1% BSA (GenView, USA) in an incubator at 37°C for 1 h and washed with PBS. Phalloidin (Invitrogen, USA) was added to the cells, and these were incubated at 25°C for 30 min. At the same time, the primary antibody was diluted (1:100) with 5% BSA in PBS solution and incubated at 4°C for 12 h. The Dylight549-labeled secondary antibody (1:100, Earthox, USA) was diluted with 1% BSA in PBS and incubated for 1 h at 25°C in the dark. DAPI (Sigma, USA) was diluted with PBS buffer to 10 µg/ml, incubated at 25°C for 5 min in the dark, and wash with PBS. The edge of the glass cover was covered with cells with filter paper to dry, the cells were placed sideways on a slide, and the slide was mounted with 50% glycerol (Sigma, USA). The fluorescence intensity of the cells was observed under a Leica DMI6000B fluorescence microscope, and images were collected. The experiment was repeated at least three times. The abovementioned cleanings were all lightly washed three times (5 min each time).

### 2.9 Statistical analysis

SPSS19.0 statistical software was used to process the data. The data used *t*-test, *χ* ^2^ test, Fisher exact probability method, and Pearson correlation test. The gray-level analysis data of protein expression was presented as mean ± standard deviation using single-factor analysis of variance. The data comparisons are *P* < 0.05, which means that the differences between the data are significant.

## 3 Results and discussion

### 3.1 Expression and localization of Girdin in diabetic brain tissue

**Figure 1:**
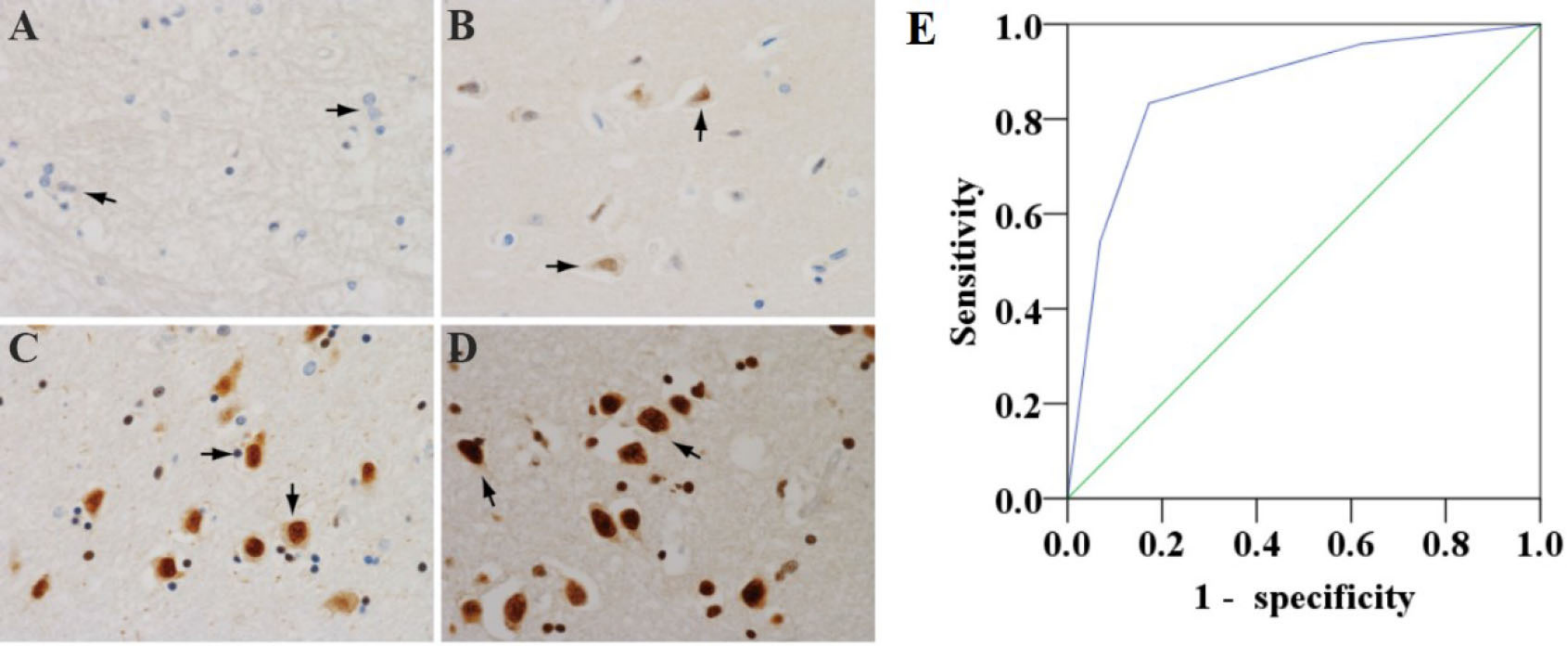
Four levels of Girdin expression in brain tissue neurons (×400). **(A)** Girdin has a low expression—brain tissue neuron Girdin does not stain. **(B)** The expression of Girdin is low, and the Girdin protein of neurons in brain tissue is stained with light yellow and the number is small. **(C)** Girdin is highly expressed. Girdin in brain tissue is stained yellow–brown, and the number is medium. **(D)** Girdin is highly expressed. Girdin in the brain tissue is stained in brown with a large number. The arrow refers to the neuron cell. **(E)** Receiver operating characteristic to evaluate the diagnostic significance of Girdin in DM patients, area under the curve = 0.863 (95% CI: 0.759–0.967).

According to the criterion of Girdin staining, the expression of Girdin in neurons was divided into four levels from – to +++ in this study (**Figure 1**). In diabetic patients, Girdin was mainly – and +, with low expression, and the low expression rate was 82.8%; in non-diabetic patients, Girdin was mainly ++ and +++, with high expression, and the high expression rate was 83.3%. The expression of Girdin in the diabetic brain tissue and the non-diabetic brain tissue was statistically obvious (*P* < 0.001), and Girdin was correlated with diabetes and was negatively correlated (*r* = –0.659, *P* < 0.001). The relationship between Girdin expression in diabetic and non-diabetic brain tissue is shown in **Table 2**. **Figures 1A–D** showed that both neurons and glial cells can express Girdin. Girdin can be localized in the nucleus alone or in the nucleus and the cytoplasm at the same time. **Figure 1E** shows the diagnostic significance of Girdin in DM patients assessed by the receiver operating characteristic, with area under the curve = 0.863 (95% CI: 0.759–0.967).

**Table 2:** Comparison of the relationship between Girdin expression and diabetic and non-diabetic patients.

Group	*n*	Girdin expression (*n*)		High expression rate (%)
		-~+	++~+++	
Diabetic group	29	24	5	17.2
Non-diabetic group	24	4	20	83.33
*χ* ^2^				20.443
*P*				<0.001

### 3.2 The relationship between the number of Girdin expression and the number of neurons

**Figure 2:**
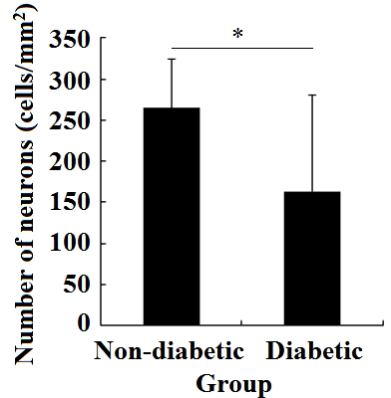
Changes in the amount of Girdin expression in the non-diabetes brain tissue and diabetes brain tissue. *P < 0.05.

The number of neurons with a high expression of Girdin in the brain tissue of diabetic patients was 163.2 ± 117.91 cells/mm^2^; the number of neurons with a high expression of Girdin in the brain tissue of non-diabetic patients was 246.4 ± 6.24 cells/mm^2^. There was an obvious difference in the number of neurons with a high expression of Girdin between the two groups (*P* < 0.05) (**Figure 2**).

### 3.3 Changes in neuronal morphology under high glucose stimulation

**Figure 3:**
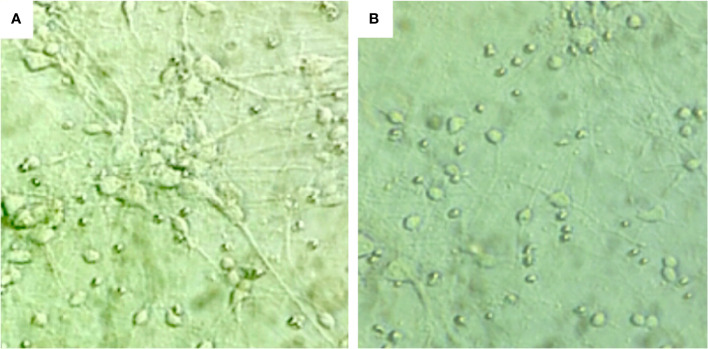
The morphological changes of hippocampal neurons under different sugar concentrations: **(A)** neuronal cells under normal conditions, ×400. **(B)** neuronal cells stimulated by high glucose (225 mM), ×400.

In the normal state of neuron cells, the neuron cell body is full, large, round, has good refraction, strongly three-dimensional, and with many cell protrusions. On the other hand, neuronal cells stimulated by high glucose (225 mM) have reduced neuron numbers, smaller cell bodies, decreased refractive index, weak stereo perception, and decreased cell synapses (branches), as shown in **Figure 3**.

### 3.4 The viability of hippocampal neuron cells decreased under high glucose stimulation and showed concentration and time dependence

**Figure 4:**
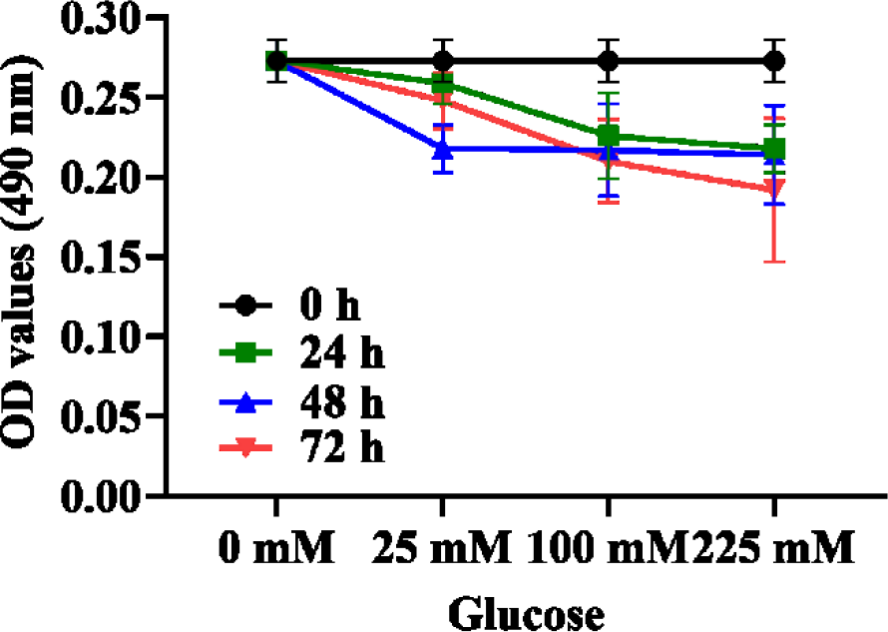
Comparison of cell viability of hippocampal neurons under high glucose stimulation.

Different concentrations of glucose (25, 100, and 225 mM) were used to treat hippocampal neuron cells for 24, 48, and 72 h, respectively, and the changes in hippocampal neuronal viability under different glucose concentrations and time were observed. The results show that high glucose stimulation can reduce the cell viability of hippocampal neurons and is time and concentration dependent (**Table 3**, **Figure 4**). At 24 h, when the glucose concentration was 100 mM, the cell viability decreased compared with the normal 25-mM-glucose group, but it was not obvious; when the glucose concentration was 255 mM, the cell viability was obviously decreased compared with the normal 25-mM-glucose group, and the difference was obvious (*P* < 0.05). At 48 and 72 h, when the glucose concentration was 100 mM, the cell viability decreased obviously, and the difference was obvious (*P* < 0.05). At 72 h, when the glucose concentration was 225 mM, the cell viability decreased most obviously. According to the results of MTT, the glucose concentrations of 25 and 225 mM for 72 h as the normal group and the high-glucose group, respectively, for the next experiment were selected.

**Table 3:** Effects of different times and concentrations of glucose medium on the viability of hippocampal neurons.

Group	*n*	Time		
		24 h	48 h	72 h
Glucose, 25 mM	12	0.259 ± 0.013	0.218 ± 0.015^a^	0.248 ± 0.018
Glucose, 100 mM	12	0.226 ± 0.027	0.217 ± 0.029^b^	0.210 ± 0.026^b^
Glucose, 225 mM	12	0.218 ± 0.015^a^	0.214 ± 0.031^a^	0.192 ± 0.045^a^

a Glucose at 225 mM in contrast to glucose at 25 mM (P < 0.05).

b Glucose at 100 mM in contrast to glucose at 25 mM (P < 0.05).

### 3.5 Apoptosis of hippocampal neurons under high glucose stimulation

**Figure 5:**
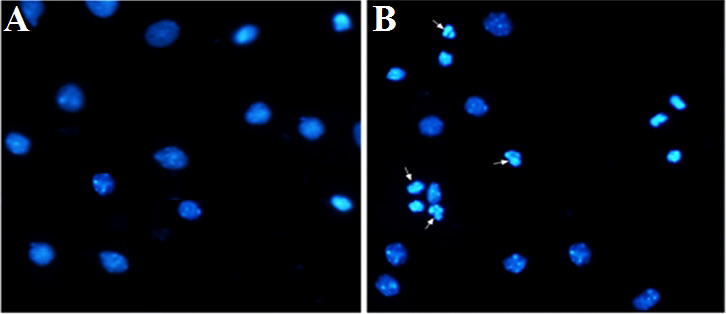
Immunofluorescence detection of the nucleus of hippocampal neurons under high glucose stimulation (×400). The arrow points to apoptotic bodies. **(A)** Glucose, 25 mM. **(B)** Glucose, 225 mM.

We used immunofluorescence to observe the nuclei of hippocampal neurons at 72 h with 25 and 225 mM glucose concentrations. The results showed that the nuclei of the neurons in the control (25 mM) group were oval or round and relatively uniform in size, while the nuclei of the neurons in the high-glucose (225 mM) group were inconsistent in size and with nuclear pyknosis, nuclear fragmentation, and apoptosis, suggesting neuronal cell apoptosis (**Figure 5**).

### 3.6 Damage to neuronal synapses under high glucose stimulation

**Figure 6:**
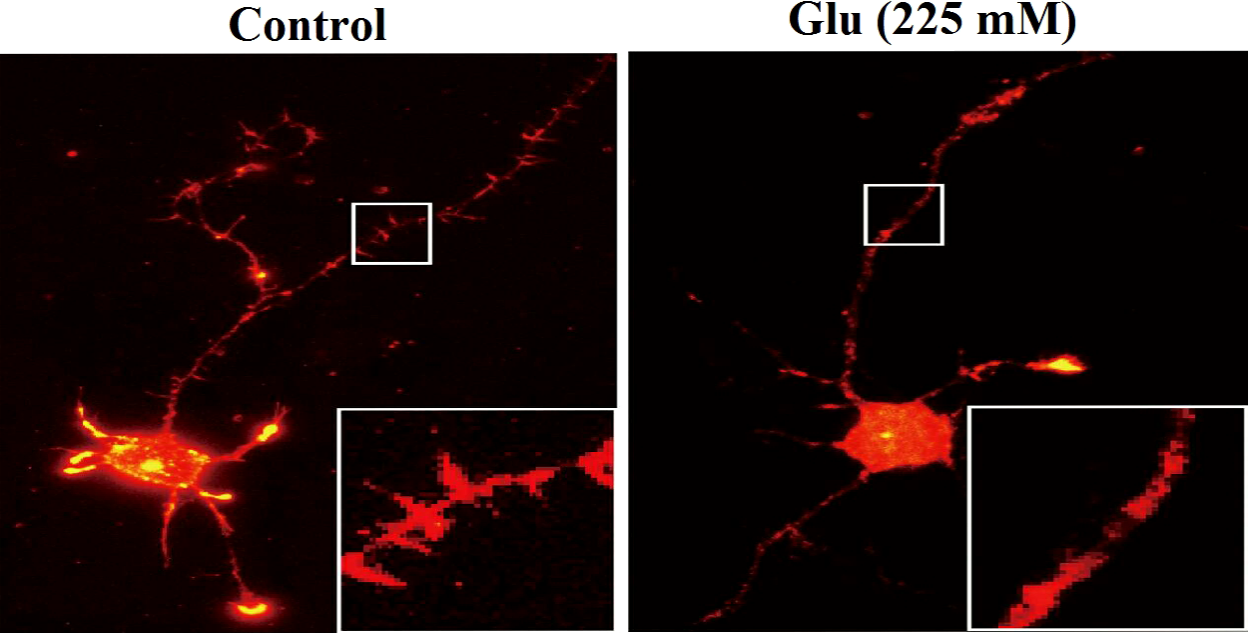
Immunofluorescence analysis of the changes of dendritic spines of neurons under high glucose stimulation (×400).

**Figure 7:**
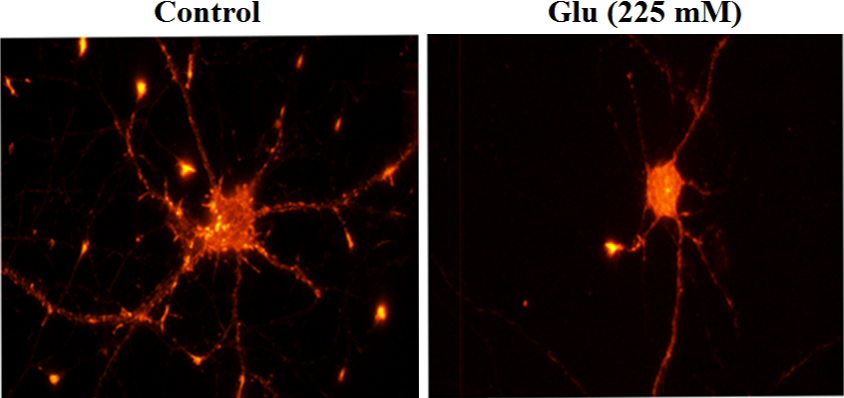
Immunofluorescence analysis of the morphological changes of neurite length and dendritic branching under high glucose stimulation (×400).

Phalloidin staining was used to observe the hippocampal neurons under the action of 25 and 225 mM glucose concentrations for 72 h. The number of dendritic spines of neurons in the high-glucose (225 mM) group was obviously less than that in the control (25 mM) group (**Figure 6**). Immunofluorescence was used to analyze the changes of dendritic branch under high glucose stimulation (**Figure 7**). The 225-mM-glucose group and the 25-mM-glucose group each randomly select 10 neuronal cells. Under the same pixel and magnification, the 225-mM-glucose group has thinner and shorter dendrites and fewer branches than the 25-mM-glucose group. The dendrite length in the 225-mM-glucose group was 342.83 ± 153.01, while the dendrite length in the 25-mM-glucose group was 487.81 ± 158.16. There was an obvious difference between the two groups (*P* = 0.016). The number of dendrite branches in the 225-mM-glucose group was 9.33 ± 4.29, and the number of dendrite branches in the 25-mM-glucose group was 20.27 ± 8.8. There was an obvious difference between the two groups (*P* = 0.000).

### 3.7 Girdin expression in hippocampal neurons decreased under high glucose stimulation

**Figure 8:**
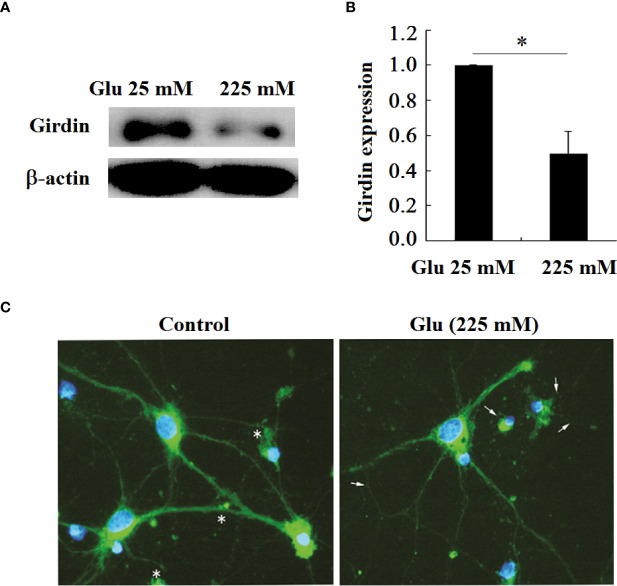
Girdin expression in hippocampal neurons decreased under high glucose stimulation, *P < 0.05. **(A)** Western blot to detect the expression of Girdin. **(B)** Grayscale analysis of Girdin expression. **(C)** Immunofluorescence analysis of Girdin expression under high glucose stimulation (×400). The arrow refers to the Girdin expression.

The previous immunohistochemical results showed that the expression of Girdin in the brain tissue of diabetic patients was reduced. In order to make a further confirmation, this study tested the expression of Girdin in hippocampal neurons under high glucose stimulation (**Figures 8A, B**). The results showed that, in contrast to the 25-mM-glucose group, the expression of Girdin in the 225-mM-glucose group was obviously reduced (*P* < 0.05). The immunofluorescence results also showed that, in contrast to the 25 mM group, the fluorescence intensity of Girdin in the neurons of the 225-mM-glucose group decreased, especially in the growth cones, synaptic terminals, *etc.* The decrease in Girdin was the most obvious (**Figure 8C**).

### 3.8 High glucose stimulates the decrease of Akt expression in hippocampal neurons

**Figure 9:**
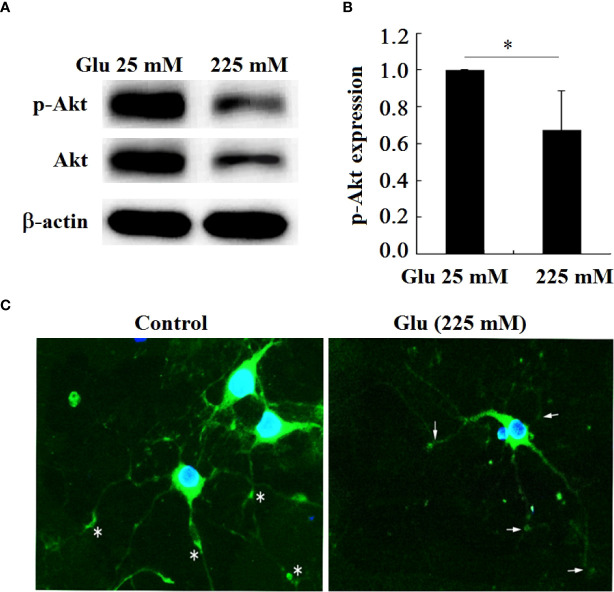
P-Akt and Akt expression in hippocampal neurons decreased under high glucose stimulation, *P < 0.05. **(A)** Western blot to detect the expression of Akt phosphorylation. **(B)** Grayscale analysis of Akt phosphorylation. **(C)** Immunofluorescence analysis of the expression of p-Akt under high glucose stimulation (×400). The arrow refers to the expression of p-Akt in the growth cone and synaptic terminal.

In order to further confirm that Girdin is related to the damage of hippocampal neurons under high glucose stimulation, this paper examines the expression of the Akt phosphorylation level of the downstream signaling molecule of Girdin (**Figures 9A, B**). The results showed that, in contrast to the 25-mM-glucose group, the level of Akt phosphorylation in the 225-mM-glucose group was obviously reduced (*P* < 0.05). Immunofluorescence showed that, in the 25-mM-glucose group neurons, p-Akt was located in the cell body and synaptic terminals, while in the neurons stimulated by high glucose, the expression of p-Akt was reduced, especially the p-Akt distributed in the synaptic terminals (**Figure 9C**).

### 3.9 The expression of STAT3 in hippocampal neurons decreased under high glucose stimulation

**Figure 10:**
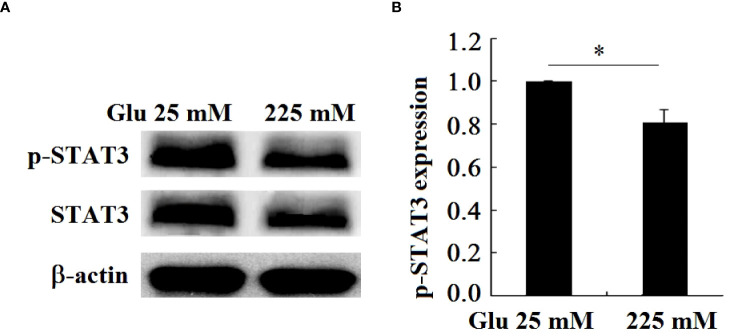
P-STAT3 and STAT3 expression in hippocampal neurons decreased under high glucose stimulation, *P < 0.05. **(A)** Western blot to detect the expression of STAT3 phosphorylation. **(B)** Grayscale analysis of the phosphorylation of STAT3.

The DNA promoter region encoding Girdin protein contains the binding sequence of STAT3, and STAT3 can initiate the expression of Girdin. In this paper, the expression of the phosphorylation level of the signal molecule STAT3 under high glucose stimulation is detected. The results showed that, in contrast to the 25-mM-glucose group, the level of phosphorylation of STAT3 in the 225-mM-glucose group was obviously reduced (*P* < 0.05). It can be seen that the expression level of Girdin in neuronal injury in a high-glucose environment is consistent with the phosphorylation level of STAT3, namely, the decrease in Girdin expression was caused by the downregulation of STAT3 (**Figure 10**).

### 3.10 Discussion

As a signal molecule, Girdin plays an important role in cell signal transduction. It can regulate cell proliferation, differentiation, migration, anti-apoptosis, and autophagy by regulating G protein, PI3k/Akt signal transduction pathway, *etc.* Girdin plays an important role in the nervous system (22–27). Girdin can act as a regulator of PI3K and G protein signaling pathways and phosphorylate Akt. The phosphorylated Akt can promote cytoskeleton rearrangement, cell proliferation, migration, and other activities. On the other hand, Akt can activate downstream substrate glycogen synthesis. Enzyme kinase-3, Forkhead transcription factor, mTOR, and various growth factors (VEGFR, EGFR, IGFR, and InsR) are involved in cell growth, proliferation, apoptosis, autophagy, and angiogenesis.

This article first analyzes the correlation between Girdin and diabetes. By detecting the expression of Girdin in diabetic and non-diabetic brain tissues, it was found that the positive expression rate of Girdin in the diabetic brain tissue and the non-diabetic brain tissue was 17.2% (5/29) and 83.3% (20/24), respectively. There was an obvious difference between the two groups (*P* < 0.05). It shows that Girdin has a low expression in diabetes. In order to further explore the mechanism of Girdin and diabetes, we used high glucose to stimulate hippocampal neurons and to simulate a neurological damage model of diabetic patients caused by hyperglycemia and observe the morphological changes and cell viability of neurons under high glucose stimulation as well as Girdin and its downstream signal molecule Akt changes. Studies have found that under high glucose stimulation, hippocampal neurons degenerate, the number of neurons is reduced, the cell body of the neuron is reduced and shrunk, and the cell synapse (branch) is reduced. The size of the neuron nucleus is inconsistent, and nucleus pyknosis and nucleus are visible. Fragmentation and apoptotic bodies appear, and some neurons are apoptotic. In the control group, neuronal cells are in good condition, with full and larger cell bodies and many cell protrusions. It can be seen that high glucose stimulation causes neuronal damage. Further observation of cell viability under high glucose stimulation showed that the cell viability decreased, and it was concentration- and time dependent. The Western blot detection results showed that, under high glucose stimulation, the expression of Girdin and Akt in hippocampal neurons was obviously reduced (*P* < 0.05). It shows that Girdin has a certain correlation with neuronal damage caused by diabetes. The results of cellular immunofluorescence further verify the neuronal damage under high glucose stimulation and the decrease of Girdin and Akt expression levels. In order to further determine the mechanism of the decrease in Girdin expression level, we also tested the level of STAT3, which regulates the expression of Girdin protein. The data showed that the level of STAT3 was obviously reduced under high glucose stimulation.

It can be seen that Girdin is closely related to neuronal damage caused by diabetes. When Girdin is missing, it will cause the migration of granular cells in the hippocampal dentate gyrus, causing cell structure damage and axon growth defects (15). The destruction and the reduction of neurons and synapses are the main manifestations of pathological changes in the brain tissue of diabetic patients. In diabetic patients, long-term chronic hyperglycemia will lead to hippocampal neuronal apoptosis and decreased synaptic plasticity (13–17). Pathological manifestations such as neuronal damage, apoptosis, and decreased synaptic plasticity and axon dendrites in the brain tissue of diabetic patients are closely related to the decrease or loss of Girdin. Moreover, as a regulator of the PI3K/Akt signal transduction pathway, Girdin can play an important role in the nervous system by activating the downstream substrate of the PI3K/Akt pathway. The PI3K/Akt signal pathway is an important signal transduction pathway closely related to cell growth and survival (26). Akt is a direct target protein downstream of PI3K. Girdin can directly bind to and activate PI3K and then activate Akt (28). Phosphorylated Akt, on the one hand, can positively feedback to Girdin, phosphorylate Girdin, and promote cytoskeletal rearrangement, cell proliferation, migration, and other activities. The PI3K/Akt signaling pathway can reduce the volume of cerebral infarction, reduce nerve cell apoptosis, and play a protective role in cerebral ischemia–reperfusion injury (29, 30). It can be seen that the expression of Girdin decreases, the PI3K/Akt signaling pathway is inhibited, and the neuroprotective effect is weakened, which will affect the growth and development of neurons, cause the destruction of neuronal structure, and ultimately lead to apoptosis. In this experiment, the expression of P-STAT3 and Girdin decreased in the injured neurons under high glucose stimulation, and the two are consistent. From this, we speculate that high glucose stimulation can cause a decrease in P-STAT3 levels, which, in turn, leads to a decrease in Girdin expression and induces neuronal damage. In summary, Girdin is closely related to neuropathy caused by diabetes. When diabetes occurs, there may be such a pathway wherein high glucose stimulation causes the level of STAT3 to decrease, which, in turn, causes the expression of Girdin to decrease. On the one hand, the decrease of Girdin causes neuronal damage by inactivating the downstream molecule Akt. On the other hand, it also can cause cell damage by causing damage to the cytoskeleton of neurons. The deficiency of this study is that no further experiments were conducted to determine changes in the phosphorylation levels of STAT3 and AKT after Girdin protein levels were rescued. Therefore, further experiments will clarify neuronal viability after Girdin protein levels, STAT3, and AKT phosphorylation levels are rescued.

## 4 Conclusion

The expression of Girdin in the brain tissues of diabetic patients decreases, and the expression levels of STAT3 and Akt related to the upstream and downstream signaling pathway molecules also decrease. It was proved that Girdin is closely related to the damage of the NRD-nervous system. In-depth research on Girdin may play an important role in revealing the pathogenesis of NRD and may also provide new ideas for the treatment of NRD.

## Data availability statement

The original contributions presented in the study are included in the article/supplementary material. Further inquiries can be directed to the corresponding author.

## Ethics statement

In this study, the wax specimen of autopsy was used, and the relevant agreement was signed before death, and complied with the 1975 Declaration of Helsinki. The study was reviewed and approved by Beijing Hospital, approval no. 2022BJYYEC-206-02.

## Author contributions

LL: methodology, investigation, data curation, and original draft. JZ: writing, review, and editing. YH: review and editing. DL: idea, supervision, review, and editing. All authors contributed to the article and approved the submitted version.

## Acknowledgments

I would like to express my gratitude to all those who helped me during the writing of this thesis. I acknowledge the help of my colleagues DL and JZ. They have offered me suggestions in academic studies.

## Conflict of interest

The authors declare that the research was conducted in the absence of any commercial or financial relationships that could be construed as a potential conflict of interest.

## Publisher’s note

All claims expressed in this article are solely those of the authors and do not necessarily represent those of their affiliated organizations, or those of the publisher, the editors and the reviewers. Any product that may be evaluated in this article, or claim that may be made by its manufacturer, is not guaranteed or endorsed by the publisher.
